# Clinimetric measurement in traumatic brain injuries

**Published:** 2014-06-25

**Authors:** N Opara, I Małecka, M Szczygiel

**Affiliations:** Jerzy Kukuczka Academy of Physical Education in Katowice, Poland “Repty" Rehab Centre in Tarnowskie Gory, Poland

**Keywords:** Traumatic Brain Injury, TBI, coma, consciousness

## Abstract

Abstract

Traumatic brain injury is a leading cause of death and disability worldwide. Every year, about 1.5 million affected people die and several millions receive emergency treatment. Most of the burden (90%) is in low and middle-income countries. The costs of care depend on the level of disability. The burden of care after traumatic brain injury is caused by disability as well as by psychosocial and emotional sequelae of injury. The final consequence of brain injury is the reduction of quality of life.

It is very difficult to predict the outcome after traumatic brain injury. The basic clinical model included four predictors: age, score in Glasgow coma scale, pupil reactivity, and the presence of major extracranial injury. These are the neuroradiological markers of recovery after TBI (CT, MRI and PET) and biomarkers: genetic markers of ApoE Gene, ectoenzyme CD 38 (cluster of differentiation 38), serum S100B, myelin basic protein (MBP), neuron specific endolase (NSE), and glial fibrillary acidic protein (GPAP).

These are many clinimetric scales which are helpful in prognosing after head injury. In this review paper, the most commonly used scales evaluating the level of consciousness after traumatic brain injury have been presented.

## Introduction

Every year, about 1.5 million affected people die and several millions receive emergency treatment because of traumatic brain injury (TBI). The costs of care after TBI are also caused by disability as well as by psychosocial and emotional sequelae of injury. It is very difficult to predict the outcome after traumatic brain injury. 

 The basic model included four predictors: age, Glasgow coma scale, pupil reactivity, and the presence of major extracranial injury. In a CT model, additional indicators were the presence of petechial hemorrhages, obliteration of the third ventricle or basal cisterns, subarachnoid bleeding, mid-line shift, and non-evacuated hematoma [**1**]. The longer the period of post-traumatic amnesia, the worse the outcome. The latter is defined as the period of time from injury until resumption of day-to-day memory. 

 There is a crude correlation between the amount of brain damage (as determined by MRI) and long-term outlook. However, there are many exceptions to these general rules and it is always unwise to give any definite prognosis within the first few weeks of injury. Most physical recovery will occur in the first 12 months, but some physical improvement can certainly occur during the second year after injury. Neuropsychological recovery takes much longer and between 2 and 3 years is usually taken as reasonable length of time for natural recovery to continue [**2**]. Following Oxford Handbook of Rehabilitation Medicine these are five factors that reduce life expectancy after TBI: immobility, incontinence, inability to swallow (necessity of PEG), on-going and uncontrolled epilepsy, severe cognitive and intellectual damage [**2**]. 

 These are many clinimetric scales that assess consciousness and disability after TBI, i.e. Glasgow Coma Scale (GCS), Grady Coma Scale, Comprehensive Level of Consciousness Scale (CLOCS), Full Outline of UnResponsiveness score (FOUR), JFK Coma Recovery Scale – Revised (CRS-R), Disorders of Consciousness Scale (DOCS), Disability Rating Scale (DRS), Coma/Near-Coma Scale (CNC), Sensory Modality Assessment Rehabilitation Technique (SMART), Rancho Los Amigos Cognitive Levels (RLA), Western Neuro Sensory Stimulation Profile (WNSSP), Sensory Stimulation Assessment Measure (SSAM), Wessex Head Injury Matrix (WHIM), Loewenstein Communication Scale (SCS), Swedish Reaction Level Scale (RLS 85), Innsbruck Coma Scale (INNS), Glasgow Liege Coma Scale (GLCS), Neurological Outcome Scale for TBI (NOS-TBI) [3-27]. In this review, the most popular and valid scales will be described. 

Scales assessing disorders of consciousness 

 The Glasgow Coma Scale (GCS) has been the gold standard of neurologic assessment for trauma patients since its development by Jennett and Teasdale in 1974 [22-24]. The GCS was found to be a simple tool to use. It became the method of choice for trauma care practitioners to document neurologic findings over time and predict functional outcome. Although the scale has been shown to be effective, many authors have cited weaknesses in the scale including the inability to predict the outcome, variation in inter-rater reliability, and the inconsistent use by caregivers in the prehospital and hospital settings. 

 GCS is based on motor responsiveness, verbal performance, and eye opening to appropriate stimuli. Individual elements as well as the sum of the score are important. The final score, expressed in the form “GCS 9 = E2 V4 M3 at 07:35", ranges from 1 to 15. Generally, brain injury is classified as severe (with GCS < 9), moderate (GCS 9–12) and minor (GCS ≥ 13). 

 According to Matis and Birbilis these are many conditions that affect the calculation of three components of GCS: ocular trauma, cranial nerve injuries, pain, intoxication (alcohol, drugs), medications (anesthetics, sedatives), dementia, psychiatric diseases, developmental impairments, no comprehension of spoken language, Intubation, tracheostomy, laryngectomy, edema of tongue, facial trauma, mutism, hearing impairments, some injuries (spinal cord, peripheral nerves, extremities), children younger than 36 months [**13**]. 

 The Grady Coma Scale classes people on a scale of I to V along a scale of confusion, stupor, deep stupor, abnormal posturing, and coma [**8**]. 

 Wijdicks et al. validated in 2005 a new coma scale: the Full Outline of UnResponsiveness (FOUR) score. It consists of four components (eye, motor, brainstem, and respiration), and each component has a maximal score of 4. Compared to GCS the inter-rater reliability was excellent with the FOUR score (kappa (w) = 0.82) and good to excellent for physician rater pairs. The agreement among raters was similar with the GCS (kappa (w) = 0.82). The FOUR score provides greater neurological detail than the GCS, recognizes a locked-in syndrome, and is superior to the GCS due to the availability of brainstem reflexes, breathing patterns, and the ability to recognize different stages of the brain herniation [**25**]. 

 In 2004, Giacino et al. have revised the JFK Coma Recovery Scale. It consisted of six subscales: auditory function, visual, motor, oromotor/verbal function, communication and arousal. Inter-rater and test-retest reliability were high for CRS-R total scores. Subscale analysis showed moderate to high inter-rater and test-retest agreement although systematic differences in scoring were noted on the visual and oromotor/verbal subscales. CRS-R total scores correlated significantly with total scores on the CRS and DRS indicating acceptable concurrent validity [**9**]. 

 In 2005, Pape et al. described a measure of neurobehavioral functioning after coma: Disorders of Consciousness Scale (DCOS). It consists of eight subscales: social knowledge, taste & swallowing, olfactory, proprioceptive & vestibular, auditory, visual, tactile and readiness. Validity analyses demonstrated that 23 of 34 test stimuli remained stable over time with no floor or ceiling effect. DOCS measures obtained within 94 days of injury predicted recovery of consciousness up to 1 year after injury (c-indices of 0.70 and 0.86). Positive (0.71) and negative (0.68) predictive values indicate that the DOCS predict recovery and lack of recovery [**15**]. 

 Rappaport et al. are authors of two scales: Coma/Near-Coma Scale (CNC, 1992) and Disability Rating Scale (DRS, 1999). The Coma/Near Coma (CNC) scale was developed to measure small clinical changes in patients with severe brain injuries who function at very low levels characteristic of near-vegetative and vegetative states. The CNC essentially expands the levels of the DRS that incorporate the vegetative and extreme vegetative categories. The CNC has five levels, based on 11 items, that can be scored to indicate the severity of sensory, perceptual, and primitive response deficits [17,18]. 

 Sensory Modality Assessment Rehabilitation Technique (SMART) has been developed in 1997 by Gill-Thwaites. It assesses eight modalities in five levels: visual, auditory, tactile, olfactory and gustatory sensation, motor functions, communication, and arousal. In 2004, Gill-Thwaites and Munday proved that SMART is a valid and reliable assessment for discriminating awareness in vegetative state and minimally conscious state [**10**]. 

 In 1989, Ansell and Keenan described the Western Neuro Sensory Stimulation Profile. WNSSP was developed to cognitively assess the function in severely impaired head-injured adults (Rancho Los Amigos levels II-V) and to monitor and predict the change in slow-to-recover patients. It consists of 32 items which assess the patients' arousal/attention, expressive communication, and response to auditory, visual, tactile, and olfactory stimulation [**3,11**]. 

 In 1994, Rader and Ellis developed the Sensory Stimulation Assessment Measure (SSAM). Patient responses on 15 items are divided into three six-point behavioral 5 subscales (visual, auditory, tactile, gustatory, and olfactory). Each item is scored based intensity on the response to non-invasive, noxious or painful stimuli in three categories: eye opening, motor and vocalization [**16**]. 

 The Wessex Head Injury Matrix (WHIM) was developed by Shiel et al. (2000) and based on previous work by Horn et al. (1992, 1993) and Wilson et al. (1994). It consists of 62 items, which are ordered in a hierarchical way, the hierarchy of behaviors assessed reflecting a statistically derived order of recovery from the coma: item 1 should appear before item 2, item 2 before item 3, etc. [**19**]. Majerus et al. showed that the WHIM scales presented good inter-rater agreement (fair to excellent inter-rater agreement was obtained for 93% of the items) and very good test-retest reliability. The study confirmed that the WHIM was largely superior to the GCS and Glasgow Liege Coma Scale for detecting subtle changes for patients emerging from the vegetative state and for patients being in a minimally conscious state. However, the study also showed that the sequence of recovery proposed by Shiel et al. (2000) is very probabilistic and lacks precision, as the proposed order of recovery could not be replicated for all items of the scale [**12**]. 

 In 2002, Borer-Alafi et al. published the Loewenstein Communication Scale. LCS measures five hierarchical functions - mobility, respiration, visual responsiveness, auditory comprehension and linguistic skills (verbal or alternative) - which are divided into five parameters and rated in developmental order on a 5-point scale by level of difficulty [**5**]. In 1984, Stanczak et al. proposed an alternative method of coma assessment compared with the Glasgow Coma Scale called Comprehensive Level of Consciousness Scale. CLOCS is a simple scale consisting of seven items assessing eye responses, motor, posture, communication and general responsiveness [**20**]. 

 In 1985, the Swedish Reaction Level Scale (RLS 85) was developed by Starmark et al. It is a single, eight grade lines scale where 1-3 means that the patient is conscious and 4-8 means that the patient is unconscious [**21**]. In 1991, Innsbruck Coma Scale (INNS) was created by Benzer et al. and modified by Diringer and Edwards in 1997. It assesses eight reactions in a four-level scoring system. The validation made in 1997 proved that both GCS and ICS are good at predicting independence (GCS and ICS, 71% correct) and mortality (GCS, 60% correct; ICS, 56% correct) and in predicting levels of outcome (help or independent) [4,7]. 

 In 1982, Born et al. developed the Glasgow-Liège Coma Scale (GLCS). It combines the GCS with a quantified analysis of five brain stem reflexes: fronto-orbicular, vertical oculocephalic, pupillary, horizontal oculocephalic and oculocardiac. The study done in 1987 showed the good agreement achieved by different examiners in the evaluation of brain stem reflexes. Brain stem reflexes offer a slightly higher agreement (kappa = 0.69) than that of the study of motor response (kappa = 0.65). In conclusion: the reliability of the evaluation of motor and brain stem parameters justifies the use of the GLCS as a means for evaluating disturbances of consciousness [**6**]. 

 Neurological Outcome Scale for TBI (NOS-TBI) has been developed in 2010 by Wilde et al. This is an adaptation of the National Institutes of Health Stroke Scale (NIHSS), specifically for clinical and research use in patients with TBI, including the addition of items specific to TBI, adjustment to the scoring algorithm to allow quantification of deficits in patients who are comatose/vegetative or agitated, and the reassignment of items (i.e., limb ataxia) that are problematic in TBI as supplemental items. The total score for the NOS-TBI is the sum of the scores for items 1–13 (based on 3-, 4-, and 5-level ratings, where 0 represents no impairment or deficit. Items 14 and 15 are considered supplemental and do not factor into the total score; thus low scores reflect less severe neurological impairment. This scale is envisioned to serve as a tool for initial stratification of injury severity, and as an outcome measure in randomized clinical trials [**26**]. 

 In 2010, Seel et al. published a report of the American Congress of Rehabilitation Medicine, Brain Injury-Interdisciplinary Special Interest Group, Disorders of Consciousness Task Force, Assessment scales for disorders of consciousness: evidence-based recommendations for clinical practice and research. According to it, the JFK Coma Recovery Scale – Revised (CRS-R) may be used to assess disorders of consciousness with minor reservations, and the SMART, Western Neuro Sensory Stimulation Profile (WNSSP), Sensory Stimulation Assessment Measure (SSAM), Wessex Head Injury Matrix (WHIM), and Disorders of Consciousness Scale (DOCS) may be used to assess disorders of consciousness with moderate reservations. The Coma/Near-Coma Scale (CNC) may be used to assess disorders of consciousness with major reservations. The FOUR, Innsbruck Coma Scale (INNS), Glasgow-Liege Coma Scale, Swedish Reaction Level Scale-1985, Loewenstein Communication Scale (SCS), and Comprehensive Level of Consciousness Scale (CLOCS) are not recommended at this time, bedside behavioral assessment of disorders of consciousness because of a lack of content validity, lack of standardization, and/or unproven reliability [**28**].

## References

[1] Perel  P, Arango  M (2008). MRC CRASH Trial Collaborators. Predicting outcome after traumatic brain injury: practical prognostic models based on large cohort of international patients. BMJ.

[2] Barnes  M, Ward  T (2005). Oxford Handbook of Rehabilitation Medicine.

[3] Ansell  BJ, Keenan  JE (1989). The Western Neuro Sensory Stimulation Profile: a tool for assessing slow-to-recover head-injured patients. Arch Phys Med Rehabil.

[4] Benzer  A, Mitterschiffthaler  G (1991). Prediction of non-survival after trauma: Innsbruck Coma Scale. Lancet.

[5] Borer-Alafi  N, Gil  M (2002). Loewenstein communication scale for the minimally responsive patient. Brain Inj.

[6] Born  JD, Hans  P (1987). Interobserver agreement in assessment of motor response and brain stem reflexes. Neurosurgery.

[7] Diringer  NM, Edwards  DF (1997). Does modification of the Innsbruck and the Glasgow Coma Scales improve their ability to predict functional outcome?. Arch Neurol.

[8] Fisher  CM (1969). The neurological examination of the comatose patient. Acta Neurol Scand.

[9] Giacino  JT, Kalmar  K (2004). The JFK Coma Recovery Scale-Revised: measurement characteristics and diagnostic utility. Arch Phys Med Rehabil.

[10] Gill-Thwaites  H (1997). The Sensory Modality Assessment Rehabilitation Technique - A tool for assessment and treatment of patients with severe brain injury in a vegetative state. Brain Inj.

[11] Hagen  C, Malkmus  D (1979). Levels of cognitive functioning In Rehabilitation of the Head Injured Adult. Comprehensive Physical Management. Downey, CA, Professional Staff Association of Rancho Los Amigos Hospital, Inc.

[12] Majerus  S, Van der Linden  M (2000). Wessex Head Injury Matrix and Glasgow / Glasgow-Liège Coma Scale: A validation and comparison study. Neuropsychol Rehabil..

[13] Matis  G, Birbilis  T (2008). The Glasgow Coma Scale - a brief review. Past, present, future. Acta Neurol. Belg.

[14] Morton  MV, Wehman  P (1995). Psychosocial and emotional sequelae of individuals with traumatic brain injury: a literature review and recommendations. Brain Inj.

[15] Pape  TL, Heinemann AW (2005). A measure of neurobehavioral functioning after coma. Part I: Theory, reliability, and validity of Disorders of Consciousness Scale. J Rehabil Res Dev.

[16] Rader  MA, Ellis  DW (1994). The Sensory Stimulation Assessment Measure (SSAM): a tool for early evaluation of severely brain-injured patients. Brain Inj.

[17] Rappaport  M, Dougherty  AM (1992). Evaluation of coma and vegetative states. Arch Phys Med Rehabil.

[18] Rappaport  M, Hall  KM (1982). Disability rating scale for severe head trauma: coma to community. Arch Phys Med Rehabil.

[19] Shiel  A, Horn  SA (2000). The Wessex Head Injury Matrix (WHIM) main scale: a preliminary report on a scale to assess and monitor patient recovery after severe head injury. Clin Rehabil.

[20] Stanczak  DE, White III JG (1984). Assessment of level of consciousness following severe neurological insult. A comparison of the psychometric qualities of the Glasgow Coma Scale and the Comprehensive Level of Consciousness Scale. J Neurosurgery.

[21] Starmark  JE, Stalhammar  D (1988). The Reaction Level Scale (RLS 85): Manual and guidelines. Acta Neurochir.

[22] Teasdale  G (1976). Assessment of head injuries. Br. J. Anaesth.

[23] Teasdale  G (1974). Assessment of coma and impaired consciousness. A practical scale. Lancet.

[24] Teasdale  G (1979). Adding up the Glasgow Coma Score. Acta Neurochir. Suppl (Wien).

[25] Wijdicks  EF, Bamlet  WR (2005). Validation of a new coma scale: The FOUR score. Ann Neurol..

[26] Wilde  EA, McCauley  SR (2010). Feasibility of the Neurological Outcome Scale for Traumatic Brain Injury (NOS-TBI) in Adults. J Neurotrauma.

[27] Wilde  EA, Whiteneck  GG (2010). Recommendations for the use of common outcome measures in traumatic brain injury research. Arch Phys Med Rehabil.

[28] Seel  RT, Sherer  M (2010). N. American Congress of Rehabilitation Medicine, Brain Injury-Interdisciplinary Special Interest Group, Disorders of Consciousness Task Force, Assessment scales for disorders of consciousness: evidence-based recommendations for clinical practice and research. Arch Phys Med Rehabil.

